# The Effectiveness of Psychoeducational Support Groups for Women With Breast Cancer and Their Caregivers: A Mixed Methods Study

**DOI:** 10.3389/fpsyg.2019.00288

**Published:** 2019-02-18

**Authors:** Sabrina Cipolletta, Camilla Simonato, Elena Faccio

**Affiliations:** ^1^Department of General Psychology, University of Padua, Padua, Italy; ^2^Department of Philosophy, Sociology, Education and Applied Psychology, University of Padua, Padua, Italy

**Keywords:** breast cancer, caregivers, effectiveness, psycho-oncology, support group

## Abstract

**Background:** Previous studies on the effectiveness of psychological interventions in oncology mainly used quantitative measures and no study was conducted with regard to both caregivers and patients.

**Aim:** This study evaluates the effectiveness of psychoeducational support groups, both for women with breast cancer, and for their informal caregivers through the use of quantitative and qualitative measures.

**Methods:** A longitudinal design was used comparing two psychoeducational support groups with other two groups in a standard care control condition. Participants were 28 women with a diagnosis of breast cancer in the care of a hospital in Northern Italy, and 21 family caregivers. The quantitative data were collected by Cognitive Behavioral Assessment for Outcome Evaluation (CBA-OE) and the qualitative data through the use of semi-structured interviews.

**Results:** The statistical analysis showed a significant change attributable to the psychological intervention that proves the effectiveness of such an intervention in the patients’ and caregivers’ group. The qualitative analysis allowed us to interpret the behavioral and psychological profile emerging from CBA-OE, by considering the subjective experience of the treatment groups. The group experience offered affective, relational and informative support, and allowed participants to create a network and to feel understood and reassured.

**Conclusion:** The results suggest the usefulness of psychoeducational support groups for women with breast cancer and for their caregivers. The value of this kind of intervention is not only at an individual level but also at a systems level, and family involvement ensures the best positive outcomes.

## Introduction

The need for psychological interventions in oncology is well recognized in the case of full-blown psychiatric disorders, in particular anxiety and depression, but sub-threshold symptoms and other aspects should not be underestimated, including the worsening of the quality of life, the negative repercussions of the neoplasia on the family group, the caregiving burden, all kinds of physical sufferings caused by collateral effects linked to treatments and, finally, the tendency of the cancer to become chronic (Anguiano et al., 2012; Gil et al., 2012; Cipolletta et al., 2013). The diagnosis and the treatment of cancer impact also on the sexual functioning and intimacy of the cancer patients, in particular breast cancer women, and their partners (Gilbert et al., 2009; Iżycki et al., 2016).

The social and psychological support of the patients and of their family members should be considered as a fundamental part of health assistance, so that neither group feels alone when facing the painful experience of neoplasia (Vanderwerker et al., 2005; Ussher et al., 2006; Badger et al., 2007; Naughton and Weaver, 2014). The interventions in the field of psycho-oncology are divided in four macro categories in the form of counseling, behavioral methods, bodily methods and psychotherapies. The final category can follow the cognitive-behavioral approach, the explorative-interpersonal approach or psychoeducational and active support intervention (Holland et al., 2015; Sanjida et al., 2018).

Psychoeducational support intervention is a global and interdisciplinary approach capable of uniting educational intervention with psychological support. The educational component provides patients and their families with adequate and realistic knowledge concerning the neoplasia, the related treatments, the collateral effects, the possible complications, and some hypotheses in terms of practical problem resolution. The psychological component deals with the affective and cognitive elaboration of the experiences related to the illness, the psychological and social process of adaptation to the cancer, the awareness of oneself and of one’s own needs, mood improvement, stress management, problem solving and coping strategies (Guo et al., 2013; Schou Bredal et al., 2014). This intervention may provide the patient with a greater feeling of control in terms of the cancer, with a consequent reduction in his or her feelings of alienation and desperation (Hutchison et al., 2006; Sautier et al., 2014). What emerges from the literature is that such intervention is suggested both for patients with a recent cancer diagnosis, for their family members, and for medical staff, in order to increase their knowledge of the matter, to render their behavior more conscious and appropriate for the situation and, at the same time, to lower feelings of disarray and of the impossibility of controlling the neoplasia (Holland et al., 2015).

In this way, patients may find it possible to develop a more active, participatory and proactive attitude toward the illness and, at the same time, may feel supported during the process of recovery (Capozzo et al., 2010; Grande et al., 2014). Several research results have demonstrated the efficacy of psychoeducational support intervention through an increase in functional coping strategies, an amelioration of the quality of life, a reduction in pain and of psychopathological symptoms (mainly depressive and anxiety related symptoms) and an enforcement of some biological parameters such as the ones linked to the immunological defense mechanisms, an amelioration of treatment compliance and the relationships with regard to the family, an increase in capacity in terms of cohabiting and facing the disease, and self-efficacy, both in oncologic patients (in particular in women with breast cancer) (Golant et al., 2003; Helgeson et al., 2006; Galway et al., 2012; Semple et al., 2013; Matsuda et al., 2014) and their caregivers (Manne et al., 2004; Leow et al., 2015).

An element which emerges from the literature is that many interventions are characterized by a group structure which is useful when it comes to providing participants with an adequate psychological support to face the devastating impact of neoplasia, to allow them to share their emotions and experiences, to elaborate on the crisis, accept the changes, and exchange with other people knowledge about practical and functional issues useful for everyday life (Holland et al., 2015). Moreover, this kind of intervention allows people to achieve a greater sense of autonomy, to be less dependent on medical staff, to use adaptive problem-solving strategies, and to develop new social ties. Patients may perceive their condition of illness differently, thanks to the feelings of relief and protection provided by the group (Gottlieb and Wachala, 2007). Furthermore, the stigmatization of the patient as an invalid may be diminished. Within the group, new energies are to be found in terms of facing cancer and its related medical treatments, and also to favor adaptation to the neoplasia through mutual confrontation; in fact, everybody gives and receives at the same time (Holland et al., 2015). Listening, confrontation and exchange with other people reduce the feelings of impotence and isolation, and increase the feelings of strength and usefulness (Chou et al., 2016). One might think that the encounter with people who are experiencing the same dramatic situation can be depressive due to the fear of contamination in terms of the other’s pain. On the contrary, participants report that the group becomes a privileged place of containment, where the expression of anxiety, rage, desperation, worries and disarray (otherwise not communicable for fear of bothering or tiring others) is allowed (Montazeri et al., 2001). Altruism represents a very important experience because the members of the group identify with each other and feel that they are useful for someone who is suffering in the same way as they are. They can talk freely about hostilities and thus obtain a feeling of relief (Chou et al., 2016).

This short review shows that previous studies have analyzed the effects of support groups on different indices of patients’ health. They mainly used quantitative measures, but some used a qualitative approach. Only two studies combined the two approaches (Montazeri et al., 2001; Mahendran et al., 2017), but no study has done this with regard to both caregivers and patients. The present study aimed to explore the effectiveness of support groups in terms of cancer patients and their family caregivers by using a specific measure developed to assess psychotherapy interventions. Moreover, qualitative data were collected in order to identify the effective factors associated with the intervention.

## Materials and Methods

### Participants

Patients and caregivers were recruited at a Public Hospital in Northern Italy. Inclusion criteria for the patients’ groups were the diagnosis of breast cancer within the previous 5 months and medical treatment in the active phase. Inclusion criteria for the caregivers’ groups were that they were the persons indicated as caregivers by the patients with a diagnosis of neoplasia in the previous 5 months.

The two treatment groups were composed, respectively, of 13 women with a mean age of 51 years (ranging from 26 to 75 years), and five caregivers (four women and a man), with a mean age of 59 years (ranging from 51 to 67 years), all of whom participated in the psychological intervention. Three caregivers were the partners of the patient, one was the mother, and one was the sister. This was the first experience of operating in a psychological support group for all participants.

The two control groups were composed, respectively, of 15 women, with a mean age of 57 years (ranging from 37 to 76 years), and 16 caregivers (3 men and 13 women), with a mean age of 48 years (ranging from 25 to 70 years), who declined to participate in the psychological intervention. Four caregivers were the partners of the patients and 12 were their adult children. The other socio-demographic characteristics of the participants of the four groups are reported in Table 1.

**Table 1:** Socio-demographic characteristics of patients and caregivers of the four groups.

	Patients				Caregivers			
		
	Treatment		Control		Treatment		Control	
				
	*N*	%	*N*	%	*N*	%	*N*	%
Married	8	62	12	80	5	100	13	81
Unmarried	3	23	0	0	0	0	3	19
Widowed	2	15	3	20	0	0	0	0
Housewife	5	38	8	53	1	20	3	19
Retired	4	31	4	27	2	40	1	6
Worker	4	31	3	20	2	40	12	75


Potential participants were identified from clinic lists that were generated by the oncological team among the patients in care at the Hospital and were invited to participate in the study. An initial sample of 32 women and 39 caregivers who met the inclusion criteria for the study was identified. Four women and 18 caregivers declined to participate because they were too busy.

Ethical principles were adhered to throughout the study. Participants were informed of their right to withdraw at any time. They signed a written informed consent form with regard to participating in the research. The Research Ethics Committee of Venezia and IRCCS San Camillo approved the study (Approval number 37/CESC).

### Measures

Cognitive Behavioral Assessment for Outcome Evaluation (CBA-OE) was used to evaluate the effectiveness of the psychological intervention. This questionnaire is composed of 80 items relating to the previous 2 weeks, which are grouped in five scales: Anxiety (A), Well-Being (WB), Perception of Positive Change (PC), Depression (D), and Psychological Distress (PD). CBA-OE has good internal consistency, good reliability, and excellent structural validity for the five interrelated dimensions (Michielin et al., 2008; Sanavio et al., 2013; Bertolotti et al., 2015).

Semi-structured interviews were conducted in order to understand experiences, knowledge, and reactions to the illness and to the group experience (Flick et al., 2004; Brown et al., 2015). On the basis of the literature review, the interview guide was developed. It was composed of twenty questions (e.g., Why did you join the group? How did you feel at the end of the group? What did the group mean for you?) and it lasted 25 min on average.

### Procedure

The group intervention was psychoeducational in nature, but the therapist also used mindfulness techniques and relaxation. The focus of the psychoeducational intervention was to provide informative, emotional and relational support to the participants during the treatment. The meetings were relatively unstructured because the topics were introduced and analyzed by the group, with the aim of helping participants explore their feelings, deal with individual concerns, and confront the fears and experiences of the participants. During the discussions, the therapist provided groups with information regarding both the medical and psychosocial aspects of the cancer. The group meetings consisted of 90-min sessions per week for a total of 12 sessions for patients’ group and six sessions for caregivers’ group over a 3-month period. The psychoanalytic therapist was a 40 years old woman, who had a containment and facilitation role in terms of group dynamics.

At the beginning of the first session, and at the end of the last session of the treatment groups, (T0–T1), each participant filled in the CBA-OE individually; at the same time the control group filled in the same questionnaire. After the conclusion of the group meetings the participants took part in individual interviews. The CBA-OE and the interviews were administered by a researcher.

### Data Analyses

The statistical analyses of the CBA-OE data were as follows:

• Independent *t*-test to evaluate the statistical significance of the mean differences between the treatment groups and the control groups at T0 and T1 for each scale;
• The effect size of the psychoeducational support intervention, in the form of Cohen’s d index (d), obtained from the comparison between the means at T0 and T1 for each scale in each group;
• repeatedly measures analysis of variance (ANOVA) according to between and within factors, which are “Condition,” “Time” and the interaction between them, “Condition^∗^Time.”

The interviews were analyzed using NVivo 10 software based on a grounded theory (GT) approach. The phases of GT analysis are open, axial and selective coding: in the first stage (open coding), hundreds of codes were obtained by identifying anchors that allow the key points of the data to be gathered. Axial coding (the second stage) brought together all the codes to create new and wider categories. Finally, selective coding (the third stage) integrated and refined the categories in order to identify a core category. The final product is a theory that is derived from data and capable of explaining investigated phenomena through systematic procedures (Strauss and Corbin, 1998; Flick et al., 2004).

## Results

### Quantitative Analysis in Treatment and Control Groups of Patients

**FIGURE 1:**
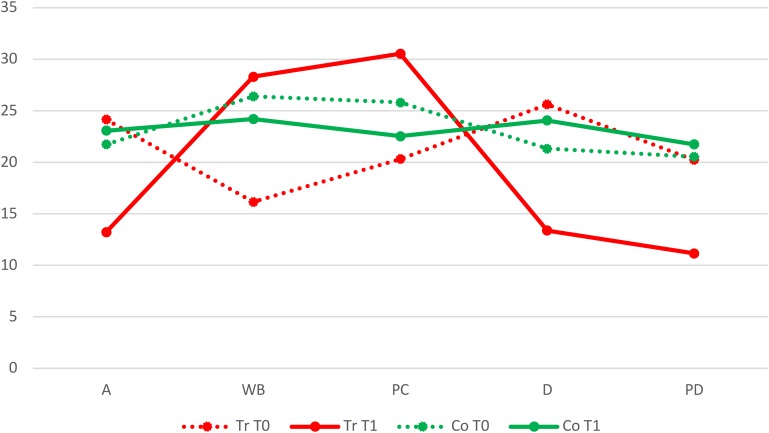
Comparison between the treatment (Tr) and control (Co) groups of patients at T0 and T1 on the Anxiety (A), Well-Being (WB), Perception of a Positive Change (PC), Depression (D), and Psychological Distress (PD) scales.

As shown in Table 2, at T0 the mean differences of the treatment and control groups in Anxiety, Depression and Psychological distress scales were not statistically significant, while in terms of Well-Being and Perception of Positive Change, the treatment group had significantly lower means. At T1 the mean differences of the two groups were statistically significant: the treatment group in comparison to the control group showed a decrease in Anxiety, Depression and Psychological Distress scales, and an increase in the perception of a Positive Change (Figure 1).

**Table 2:** *T*-Test comparisons between the treatment and control groups of patients and caregivers at T0 and T1.

	Patients						Caregivers					
						
	Treatment		Control		*T*	*P*	Treatment		Control		*T*	*P*
								
	*M*	*SD*	*M*	*SD*			*M*	*SD*	*M*	*SD*		
A T0	24.154	9.940	21.733	11.228	0.600	0.554	23.800	10.918	17.563	10.614	1.140	0.268
A T1	13.231	5.674	23.067	11.023	-2.897	0.008	14.800	8.319	21.688	11.306	-1.251	0.226
WB T0	16.154	9.780	26.400	6.445	-3.316	0.003	18.200	10.134	25.188	11.508	-1.214	0.240
WB T1	28.308	9.259	24.200	10.129	1.113	0.276	28.200	14.464	22.313	12.208	0.904	0.377
PC T0	20.31	6.382	25.800	7.321	-2.099	0.046	21.800	4.658	24.375	4.031	-1.205	0.243
PC T1	30.538	7.043	22.533	6.151	3.212	0.004	25.200	4.087	21.500	5.164	1.457	0.161
D T0	25.615	14.027	21.333	11.011	0.904	0.374	19.800	8.643	17.188	8.635	0.590	0.562
D T1	13.385	4.292	24.067	13.161	-2.794	0.010	12.200	8.928	23.500	8.996	-2.456	0.024
PD T0	20.231	12.584	20.533	13.076	-0.062	0.951	11.200	6.221	15.438	10.178	-0.872	0.394
PD T1	11.154	4.688	21.733	13.874	-2.617	0.015	9.600	6.427	20.875	10.475	-2.254	0.036


In the treatment group, in contrast with the control group, the effect size was large in all scales. In particular, in the Anxiety (*d* = 1.350), Depression (*d* = 1.179) and Psychological Distress (*d* = 0.956) scales, the indexes had a positive sign, because the means decreased from T0 to T1, while in Well-Being (*d* = -1.276) and Perception of Positive Change (*d* = -1.522) scales, there was a negative sign, because at T1 the means had increased.

ANOVA (Table 3) did not show a statistically significant effect in terms of the Condition factor, while there were significant effects both of the Time factor and the Condition^∗^Time factor in five scales: Anxiety, Well-Being, Perception of Positive Change, Depression, and Psychological Distress.

**Table 3:** ANOVA calculated for treatment and control groups of patients and caregivers.

		Patients		Caregivers	
			
	Factor	*F*	*P*	*F*	*P*
A	Condition	1.210	0.281	0.004	0.952
	Time	9.029	0.006	3.084	0.095
	Cond.^∗^Time	14.749	0.001	22.355	<0.001
WB	Condition	1.154	0.293	0.009	0.925
	Time	7.245	0.012	2.698	0.117
	Cond.^∗^Time	15.067	0.001	8.811	0.008
PC	Condition	0.300	0.589	0.065	0.801
	Time	9.574	0.005	0.102	0.753
	Cond.^∗^Time	35.964	<0.001	14.619	0.001
D	Condition	0.685	0.415	1.022	0.325
	Time	6.308	0.019	0.216	0.647
	Cond.^∗^Time	15.660	0.001	25.260	<0.001
PD	Condition	1.731	0.200	2.666	0.119
	Time	5.306	0.030	2.102	0.163
	Cond.^∗^Time	9.032	0.006	7.069	0.016


*All the correlation indices are significant (*p* < 0.05).*

### Quantitative Analysis in Caregivers’ Treatment and Control Groups

With regard to the *t*-Test (Table 2), in all scales at T0 the mean differences of the treatment and control groups were not statistically significant but, in contrast with the control group, the means of the treatment group showed worse conditions, because Anxiety and Depression scales were higher while the Well-Being and Perception of Positive Change scales were lower. At T1 the mean differences of the two groups were statistically significant only in the Depression and Psychological Distress scales, where the means were lower in the treatment group. Beyond the statistical evaluation, according to clinical evaluation, at T1 there was an inversion of initial conditions because in the treatment group, unlike the control group, the means of the negative scales were decreased, and the means of the positive scales were increased.

In the treatment group, in contrast with the control group, the effect size was large in the Anxiety (*d* = 0.927), Well-Being (*d* = -0.801) and Depression (*d* = 0.865) scales, medium in the Perception of Positive Change (*d* = -0.776) scale and, finally, small in the Psychological Distress (*d* = 0.253) scale. The effect size was variable, but the signs confirmed the positive change associated with the intervention, because the signs in the negative scales were positive, thanks to the mean decrease, while in the positive scales they were negative, thanks to the mean increase.

In ANOVA (Table 3) the Condition and Time factors, individually taken, did not have any significant effect, while the Condition^∗^Time factor had a statistically significant effect in all scales.

### Qualitative Analysis

The generative research question was “What happens inside the group in terms of the experience of participation?” The open coding produced a list of labels and *in vivo* codes (referred to in parentheses) that in the axial coding were grouped into five main categories through the selection of themes that were salient and recurrent in terms of density, frequency and intensity. The main categories were linked together and overlapped in both groups.

*Motivations* included curiosities, needs, expectations and fears (conditions for the group), that brought the participants to the group, such as the possibility of attending the group for the first time, receiving information on disease and treatments, to compare their experiences with other patients or caregivers, and receiving psychological help.

*Group experience* included how the participants became part of the group (being referred), what they experienced during sessions both in terms of informative aspects, thanks to the realization of possibilities and the development of specific issues, and affective-relational aspects, thanks to the establishment of rapport with other participants (group’s resources), and, finally, what they hoped for in terms of future groups after identifying problems and the possible suggestions made by their group (rethinking of the group).

Moreover, participants represented the intervention using an image of a single line with, on the one end, the initial negative condition, both physically and psychologically and, on the other end, at the conclusion of the group the physical and psychological improvement or the maintenance of previous conditions (beginning to end), taking into consideration the present moment and the future (after the group’s dissolving).

*Interpersonal relationships* involved all the people who directly, for example the therapist and other participants (in the group), or indirectly, for example family, friends, acquaintances and doctors (outside the group), took part in the psychological intervention and the medical treatment. The relationship between the participants was identified as that of friendship, while the therapist demonstrated professionalism and humanity. Families and friends showed two attitudes: some showed difficulties in accepting and talking about disease, and skepticism toward the group, others showed support during treatment and participated in the group. Doctors’ negative communications, emotional detachment and lack of comfort prompted them to participate in the group.

*Group evaluation* indicated the great potential of the group, because it was compared to a fundamental and indispensable experience, an enrichment, a cure, and a form of medicine during the medical treatment. The participants indicated the positive and useful role of the intervention (results) thanks to changes on a personal and social level to a greater of lesser extent (changes).

**FIGURE 2:**
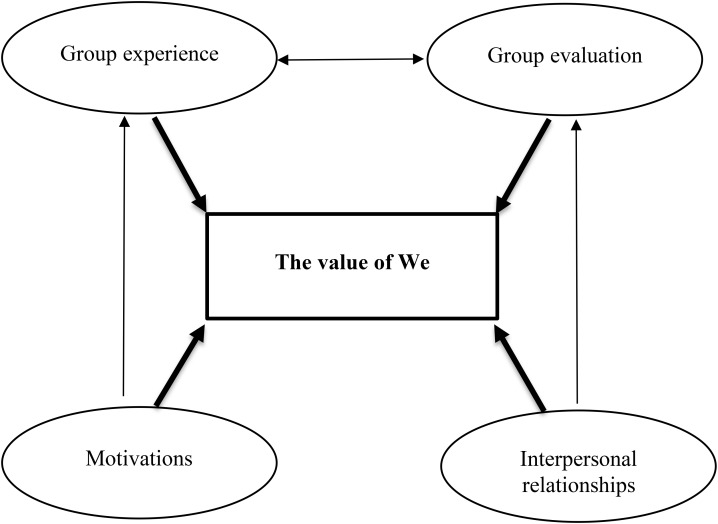
Graphic representation of the theory emerging from interviews.

Through the use of selective coding it has been possible to define the core category “The value of We,” as indicating the relationship among the macro-categories and, consequently, the theory (Figure 2). The term “We” indicates all the individuals, both from inside and outside the group, who have supported the patients and the caregivers in this difficult period of their lives, such that associated with the cancer diagnosis and the consequent therapies.

## Discussion

From the analysis of the results a significant change is apparent in the group of patients who participated in the intervention. This change can be attributed to the participation in the psychoeducational group and can be considered evidence of the effectiveness of such an intervention. These data confirm the results of previous studies pointing out that psychoeducational support intervention targeting women affected by breast cancer have the effect, on the one hand, of reducing anxiety, depression, fatigue, intrusive thoughts related to the neoplasia, feelings of confusion, uncertainty, rage and negative coping strategies and, on the other hand, support an increase in the patient’s adaptability, their sense of controlling the cancer, of emotional functioning, of interpersonal relationships, of their health status, and finally of obtaining adequate information concerning neoplasia, the collateral effects of medical treatments, body image and coping strategies (Montazeri et al., 2001; Dolbeault et al., 2009; Park et al., 2012).

From the analysis of the results with regard to the caregivers’ treatment group, a significant change emerges due to the participation in the psychological intervention, which has been proved to be effective, even if in a less evident way when compared with the patients’ group. These data are also reflected in the literature, given that psychoeducational support interventions targeting the caregivers of oncologic patients seem to be useful when it comes to comparing different situations, and have the effect of normalizing lived painful experiences: they seem to increase the coping strategies, the self-efficacy, the social functioning, the perception of well-being and the quality of life, to reduce the caregivers’ burden, the distress, anxiety and depressive symptoms, and to satisfy their need for information. Finally, they seem to help the development of relationships inside and outside the oncologic patient’s family (Bultz et al., 2000; Northouse et al., 2010; Mahendran et al., 2017).

The qualitative analysis of the interviews shows that the effectiveness of such intervention can be attributed to the group dimension. The psychoeducational support groups have been the means to create a network, the key points of which have been the patients, the caregivers, the therapist, the medical staff, the general practice doctors, the relatives, the friends, the acquaintances and charities. Thanks to the connections and the relationships among all these subjects, a synergy has been developed that allowed the therapist to create and develop several sessions based on informative and affective-relational aspects. The “Value of We,” which represents the core category that emerged from the GT analysis, is greater than the sum of its parts, and it has underlined the importance of a plurality of people that, in spite of their different objectives and roles, have joined together for a common aim, represented by the support of the participants. The network has provided both the patients and the caregivers with certainty and security and the *fil rouge* has been represented by the support that joined all the members of the network in a sort of reciprocity, since every member was, at the same time, both the source and the beneficiary of the help.

The group dimension of the psychoeducational support interventions guarantees affective support in the face of the devastating impact of cancer, the elaboration of the crisis, the adaptation to the condition connected to the neoplasia, the sharing of emotions and experiences, the exchange of information and practical details with regard to everyday life, the development of new social relationships that alleviate the sense of isolation and loneliness, the discussion of effective problem solving strategies and, finally, the increase of personal resources, both for the healing and recovery process, and for improvement in the quality of life (Cameron et al., 2007). The group is a privileged and protected micro-cosmos where the participants feel understood and reassured as a result of the sharing and the relief of the people who live in the same condition; it allows change, experiences of interpersonal learning and interaction (Montazeri et al., 2001). The group represents a therapeutic opportunity for the participants thanks to the affective-relational and informative aspects that intertwine, and which allows them to perceive the pathology as an opportunity for a significant existential improvement, giving a new order to the properties and finding an authentic sense of self and one’s own affections (Mahendran et al., 2017).

Following the literature, it is possible to identify several aspects of psychoeducational support intervention that are functional in terms of improving the quality of life. First, the intervention promotes a psychological sense of belonging to the group, given that a cancer diagnosis can lead to a lack of relationships, both from a purely numerical point of view and in terms of reliability, and to feelings of loneliness; inside the group the presence of people that are going through the same experience and sharing the same problems, alleviates the loneliness, such that the subject feel that they are part of a new community, thanks to such a close network of social relationships (Cipolletta et al., 2018). The opportunity is offered to express difficulties and to experience a sense of catharsis, since expressing, sharing and knowing problems and feelings, but also hopes, generates a sense of liberation and provides a concrete psychological support. The management of stress, due to the knowledge of the causes of the neoplasia, as well as of different possible reactions and applicable strategies, favors health education through the promotion and protection of well-being. After the upset provoked by the cancer diagnosis, the intervention allows people to change their role models by redefining competences, behaviors and lifestyles. Listening to other people’s experiences allows the patients to discover possibilities for improvement, and stimulates the development both of effective coping strategies for the problems of their daily lives and of general positive attitudes concerning their adaptation to the neoplasia and, more generally, with regard to their existence (Docherty, 2004). Inside the group the role of those who offer help and of those who receive it is interchangeable, according to the principle of equity and the capacity to offer assistance. In particular, taking part improves self-esteem, the sense of competence and responsibility, and the awareness of being useful and stimulating the abilities of the others in the group (Cipolletta et al., 2017). As far as mutual support is concerned, the mere quantity is not as important as its perception, and this represents a very good predictor both of the physical and psychological well-being of the individual and the capacity to adapt to new stressful situations (Mahendran et al., 2017).

The group dimension did not prove to be the most appropriate when it came to exploring the fertility and sexual problems of women with breast cancer. We do not know if this might depend on the psychotherapeutic approach of these specific groups, or on women’s opinion that these topics are too personal and intimate for group discussion. Neither groups of caregivers discussed these topics, but in this case the heterogeneity of the group might also have had an impact, because not all the members were the patients’ partners. Other studies have explored the experience of the partners of cancer patients with particular reference to sexuality and intimacy, and highlighted the fact that partners accept the diminishment in their sexual relationships, but express feelings of disappointment, anger and sadness about this loss (Gilbert et al., 2009). As regards fertility loss, Vitale et al. (2017) underlined its negative impact on sexual function and on psychological wellbeing, and the importance of fertility preservation techniques for the improvement of quality of the life of women who survive breast cancer. Given the importance of fertility and sexual issues, psychoeducational interventions covering these areas should also be implemented, and future studies might explore their effectiveness.

This research has a number of limitations, particularly the small number of participants in the clinical and control groups and the different sizes of the groups. Nevertheless, in combining qualitative and quantitative analyses, this study represents an attempt to highlight some features of a psychoeducational support group intervention, both for the cancer patients and their caregivers, which has proved to be useful. These results underline the importance of considering psychological and social factors in the patients’ and caregivers’ process of adaptation to the neoplasia, in the recovery process and, finally, in the promotion of health.

## Ethics Statement

This study was carried out in accordance with the recommendations of APA guidelines with written informed consent from all subjects. All subjects gave written informed consent in accordance with the Declaration of Helsinki. The protocol was approved by the Research Ethics Committee of Venezia and IRCCS San Camillo.

## Author Contributions

SC designed the study. EF contributed to the conception of the study. CS collected the data. SC and CS analyzed the data. SC, CS, and EF wrote the manuscript. All authors contributed to manuscript revision, read and approved the submitted version.

## Conflict of Interest Statement

The authors declare that the research was conducted in the absence of any commercial or financial relationships that could be construed as a potential conflict of interest.
